# Do managers sleep well? The role of gender, gender empowerment and economic development

**DOI:** 10.1371/journal.pone.0247515

**Published:** 2021-03-17

**Authors:** Xiao Tan, Leah Ruppanner, David Maume, Belinda Hewitt

**Affiliations:** 1 School of Social and Political Sciences, The University of Melbourne, Melbourne, Victoria, Australia; 2 Department of Sociology, University of Cincinnati, Cincinnati, Ohio, United States of America; Universitat de Valencia, SPAIN

## Abstract

Work demands often disrupt sleep. The stress of higher status theory posits that workers with greater resources often experience greater stress. We extend this theory to sleep and ask: do managers report more disrupted sleep and does this vary by gender and country context? Data come from the 2012 European Social Survey Programme and our sample comprised those currently employed in their prime working age (n = 27,616; age 25–64) in 29 countries. We include country level measures of the Gender Development Index (GDI) and gross domestic product (GDP). We find that workers sleep better, regardless of gender, in countries where women are empowered. For managers, women sleep better as GDI increases and men as GDP increases. Our results suggest that men experience a sleep premium from economic development and women from gender empowerment.

## Introduction

Sleep is increasingly understood as another form of inequality connected to our waking lives. The literature on sleep and work can be divided into two broad streams. The first identifies the ways in which work demands impinge upon sleep. Work time has a strong negative impact on sleep [1], in part because longer work time detracts from time available for sleep [2]. Subjective work experiences also linger into sleep. Negative experiences at work often result in poorer sleep quality [3]. Sleep complaints are also higher for those who have physically strenuous working conditions, psychosocial job strain and work-family conflicts [4, 5]. This literature draws a clear link between sleep and work, illustrating that work time, stressful work experiences and sleep are intimately connected. Yet, absent from this research is whether those who face greater workplace demands—managers—experience worse sleep.

Another stream of sleep research focuses on how sleep differs by gender. The bulk of this work focuses on the gendered distribution of family demands and sleep. This literature shows that sleep is socially patterned by gender, with children and spouses more likely to disrupt women’s than men’s sleep [6]. Women cite men’s need to be their best at work as a key justification for protecting men’s sleep [6]. In this regard, traditional breadwinning norms structured couples’ sleep patterns with women giving more value to men’s rest [7]. Other studies have focused on how gender and work intersect to structure sleep. Most focus on non-standard work to show sleep quality of working women is disturbed by both work and family interferences [8]. Mothers working night-shifts often synchronize sleep with children’s schedules which ultimately reduces their total time in rest [9] and report higher stress as a result [10]. Others show that having unpaid caregiving roles adds further burden to women care workers, resulting in poorer subjective sleep quantity and quality [11]. As this robust literature demonstrates, sleep is socially patterned by gender, work and family. Here, we extend this work to test whether women managers, who should have higher levels of job control but also more demands than other employees, report more restful sleep.

To conceptualize these relationships, we draw upon the stress of higher status theory that posits workers in higher status, more demanding jobs experience greater stress and strain [12]. We extend this theory to examine sleep and hypothesize that managers will have worse sleep than other employees even though they have greater power, control and resources. Drawing upon cross-national data, we also test whether living in a country where women hold more power and economic resources are higher, ameliorates some of this negative effect. In this way, we build upon emerging research showing everyone sleeps better in more gender equal nations [13]. Whether these benefits extend to those who are employed, and to managers in particular, is explored here.

We apply data from the most recent wave (2012) of the European Social Survey (ESS) with a sleep quality measure. These data are paired with two country-level measures—the Gender Development Index (GDI; Source: United Nations Development Programme [14]) and Gross Domestic Product (GDP; Source: United Nations Development Programme [14])–available for 29 countries in the 2012 ESS. We restrict our sample to those who are currently employed and in their prime working age (25 to 64 years). We estimate multilevel logit regression models to account for the nesting of individuals within countries. Our results indicate that sleep is patterned by managerial status, gender empowerment and economic development, with important differences by gender.

## Literature review

### Gendered expectations in sleep, work and family

Sleep is intimately connected to structures of gender, work and family life. Employment can have a negative impact on sleep, whether through hours worked [13] or workday stress disrupting nighttime sleep schedules [14]. Traditional gender roles and expectations also structure sleep, as men’s breadwinner status often elevates their right to restful sleep [6] with men more likely than women to perceive sleep as vital to supporting the family [15]. Irrespective of their employment status, mothers generally carry larger responsibilities in housework and childcare [16] and subsequently experience disrupted sleep due to family-related stress [15, 17]. Time inevitably becomes a valuable, but finite, resource through which workers must leverage expectations, demands and values to produce positive outcomes in competing elements of their life, frequently at the cost of sleep.

Competing expectations of work and family permeating into workers’ sleep is consistent with role strain theory [18] and there has been significant literature highlighting the links between workplace characteristics and workers’ experience of inter-role strain [19, 20]. The stress and mentally taxing demands of working life has significant effects on sleep. Existing research has linked employment with sleep through variables such as work hours, financial concern and employment status [2, 14, 21]. However, notably absent from the extant research literature is a detailed and nuanced understanding of which workplace characteristics and roles are most important for sleep.

### Workplace demands and resources: The stress of higher status theory

In particular, this paper is concerned with examining the sleep impact of being in a management position at work and whether this differs for men and women. There are good reasons to think managers’ sleep would be different than non-managers with important gender differences. To understand these relationships, we draw upon the stress of higher status framework. The stress of higher status theory draws upon the job demands and resources (JDR) perspective [12]. Rooted in the organizational literature, the JDR theorization is simple: jobs with greater demands are primarily related to feelings of exhaustion and jobs with greater resources facilitate a range of good individual outcomes [22]. The stress of higher status view extends this theoretical approach by identifying how well-resourced high-status jobs can contribute to stress: in particular, workers with higher earnings are more frequently exposed to interpersonal conflict and work-to-home interference [23, 24]. In addition, the stress of higher status view challenges the common assertion that individuals with more work-related resources attain work-home balance more easily; on the contrary, high status workers may transform some elements of work-related resources into demands and experience more work-nonwork interference as a result [12]. Thus, conceptualizing job demands as negative and resources as positive is too simplistic—high status jobs can have resources that increase, rather than alleviate, stress.

Empirical evidence supports these claims, as employees in jobs with higher resources—managerial, and professional workers—report greater work-family strain, more stress, and poorer emotional well-being [24, 25]. High status workers reported more work-to-home conflict especially among those working longer hours, with more authority, greater demands and more involvement [26]. It seems reasonable to expect that those in higher status positions, here managers, are more likely to carry these stressors into their sleep. Yet, no study to our knowledge tests these theoretical tensions for sleep to see if workers in high status jobs—notably, managers—experience more restless sleep and whether these experiences vary by gender and country-constraints. Understanding these relationships is important from an economic and health perspective as lack of sleep has a strong negative relationship with productivity [27, 28]. Further, these experiences are gendered, meaning women managers’ sleep will be even more disrupted due to reasons outlined below.

### Persistent gender inequality in management?

Globally, women are under-represented in management positions across most industries and sectors [29] which has been attributed to a range of factors including gender stereotypes, unconscious bias and work family responsibilities [30–32]. Women are often viewed as being less committed to their work and lacking ambition [33]. Also, their career paths often do not match the traditional expectations of linear career trajectories [32]. As a consequence, enhancing gender representation in management has been a central indicator of broader gender inequality across nations.

Regardless of gender egalitarianism in contemporary society, many sectors of employment around the world still abide by a simple ethos in “think manager, think male” [12, 34]. Women in management are expected to hold skills and express behaviors that are believed to be inconsistent to their essentialist nature as women [35, 36]. As such, while more women entered into management, they tended to work in post-industrial industries that emphasize care work (such as health, education, and welfare) and service provision. Conversely, men still occupy authority and managerial positions in “older” and industrial sectors, including construction, engineering, finance and manufacturing [37, 38]. In addition, there remains a substantial earnings penalty for managers who work in women-dominated occupations [39].

The work demands of managers are significantly shaped by gendered expectations and cultural stereotypes surrounding family life. Blair-Loy [40] found that women executives in the U.S. are expected to be focused on both work and family. This was in sharp contrast with the expectations for men, who had no “competing devotions” between work and family and allowed for a singular focus when making career choices. While at work, women managers also experience the workplace differently to their men counterparts. Women managers face more challenges in claiming managerial authority. Based on interviews with women managers in Finland and Scotland, Jyrkinen and McKie [41] highlighted their experiences of not being taken seriously at a younger age due to perceived inexperience, and questioning of their dedication to the workplace if they are single due to the expected eventuality of marriage and child-rearing.

Collectively, this literature suggests that women managers continue to face barriers tied to their gender. This differential experience may add stress to women managers’ workdays which may disrupt sleep. Here, we test these assumptions by looking at whether managers report worse sleep than those in lower positions paying careful attention to gender differences in these experiences. Our results indicate that women managers are disadvantaged in sleep but with important cultural differences by gender empowerment and economic development.

### Country-level constraints: Gender and economic development

Drawing upon existing literatures, we expect workers to experience different sleep quality based on their managerial status and gender. Yet, these experiences may also be structured by resources available in their national contexts. We focus here on two dimensions—gender development and gross domestic product. Our expectations are outlined in more detail below.

#### The gender development index

For many European countries, elevating women’s status by reducing structural barriers has been a primary goal. The United Nations identified gender inequality as a key barrier to human development and has been measuring women’s status as a key component of the human development report for 25 years [42]. We draw upon a larger literature showing gender development at the country-level is associated with greater gender equality at the individual-level. The bulk of this research focuses on unpaid domestic labor to show women in countries with greater gender empowerment spend less time in and have more equal divisions of housework [43–45]. Men also contribute a larger share of childcare in more gender empowered countries and women are less likely to tolerate unequal divisions as fair [46–48]. Simply, countries with higher levels of gender empowerment have greater gender equality at the individual-level.

An emerging literature shows gender equality is also good for health. Dahlin and Harkonen [49] illustrate the narrowed gender gap of self-reported health in countries such as Finland and Great Britain when compared to Eastern and Southern European countries. While no association is found between the UN Gender Inequality Index and gender differences in self-reported health, women report improved health outcomes in nations with greater rates of human development [49]. The benefits of gender equality are better demonstrated in mental health outcomes, with both men and women in countries with higher UN gender empowerment scores reporting lower levels of depression in the European Social Survey [50]. Similarly, Hopcroft and Bradley [51] highlight that women have higher rates of self-reported depression in the World Values Survey data of 29 countries, but the gender gap within nations is smaller in those countries with greater gender egalitarianism. Couples also sleep more restfully in countries with greater gender equality, regardless of gender or their domestic loads [13].

Collectively, this literature indicates that gender equality at the institutional-level brings a range of health rewards to citizens, including more restful sleep. In this paper, we expand upon these literatures to understand how gender development at the country level structures sleep for a key group—the employed—paying careful attention to gender and managers who have the greater resources and demands.

#### Gross domestic product (GDP)

Given our focus on the employed, we also examine the impact of economic development on employee’s sleep quality. Economic development could structure workers’ sleep in multiple ways. On the one hand, workers may sleep more restfully in countries with higher economic development as jobs and economic resources may be more bountiful and the economies may be more stable. On the other hand, living in a country with higher GDP may intensify strain and deteriorate sleep as pressures around economic performance may be greater. We directly test for these effects.

The current literature is still limited and inconclusive. Among the existing studies, many focused on the sleep impact of economic crisis. For example, Asgeirsdottir, Corman, Noonan, and Reichman [52] studied the health-related changes during the 2008 economic crisis in Iceland and showed that sleep increased during the crisis and declined during the recovery supporting the latter contention that pressure for economic productivity and recovery might be bad for sleep. Also related to the 2008/2009 Global Financial Crisis (GFC) in the United States, people reallocated a large fraction of their decreased work time toward sleep [53]. However, a study of the Michigan Recession (2008/2009 GFC) suggested that associations between material hardship and negative sleep outcomes were not statistically significant after adjusting for a robust set of sociodemographic and health characteristics [54]. Another study of an earlier economic recession in Finland [55] also concluded that the sleep quality of the general population did not drastically deteriorate during the recession.

Others focused on the link between macro-level economic conditions and individual-level sleep outcomes. A study of American adults [56] showed that higher aggregate unemployment was associated with longer mean sleep duration. Focusing on the Australian population, Perales and Plage [57] similarly found that individuals who live in areas with high unemployment rates sleep less than comparable individuals. More recently, Niekamp [58] found that economic conditions have differential impacts: economic expansions decreased weekday sleep but increased weekend sleep. These results are mainly driven by individuals most susceptible to economic fluctuations: minorities, single adults, and individuals with less education.

Although these literatures are mixed, we expect aggregated economic growth (GDP) to be positively associated with workers’ sleep as economies are more stable and economic resources more bountiful. We extend the findings of Niekamp [58] by hypothesizing that there are gender differences in the sleep impacts of economic conditions.

## Hypotheses

From these literatures, we draw three hypotheses.

*Stress of Higher Status Hypothesis*:

H1: Mangers will report poorer sleep quality than those in non-managerial positions
H1a: Women managers will report poorer sleep quality than men managers.

*Gender Empowerment Hypothesis*:

H2: The gendered sleep gap for employees and managers will narrow in countries with greater gender development.

Economic Development Hypothesis

H3: Employees in countries with higher GDP will sleep better than those in countries with weaker GDP.
H3a: The positive sleep benefits of GDP for employees will be stronger for men than women and men than women managers.

## Materials and methods

### Data

This study combined individual-level data from the 2012 European Social Survey (ESS) and 2012 macro-level data from the United Nation’s Human Development Data (1990–2018). Both datasets are publicly available and our data were downloaded from their websites (ESS: https://www.europeansocialsurvey.org/data/download.html?r=6; Human Development Data: http://hdr.undp.org/en/data#). The 2012 ESS was used because it has the most recent data available on sleep and other personal and social wellbeing factors.

We selected those who are employed in their prime working age (25 to 64) and who were complete on reports of occupation group, work time and sleep. As imputing data does not account for the nested structure of multilevel models and thus violates the assumptions of multilevel models at the country level [59, 60], we also drop those who failed to report their income.

Our final analytical sample comprised 18,116 observations (8,949 female and 9,167 male respondents) across 29 countries: Albania, Belgium, Bulgaria, Cyprus, Czech Republic, Denmark, Estonia, Finland, France, Germany, Hungary, Iceland, Italy, Ireland, Israel, Kosovo, Lithuania, Netherlands, Norway, Poland, Portugal, the Russian Federation, Slovakia, Slovenia, Spain, Sweden, Switzerland, Ukraine, and the United Kingdom.

### Dependent variable

Sleep quality was assessed by a single item that specifically asked about sleep. Respondents were asked how much of the time during the past week their sleep was restless. They answered on a four-point ordinal scale ranging from 1 (none or almost none of the time) to 4 (all or almost all of the time). This measure was chosen for several reasons. First, it is the only question specifically asking about sleep in ESS. More importantly, such a simple Likert-style rating of sleep quality is a relatively standard measure of sleep quality in the public health literature [61]. Similar measures have been widely applied as the key dependent variable in previous studies [13, 62–64].

The responses were skewed: 48% and 39% of the respondents reported that they had restless sleep none of the time or sometimes, while 10% and 4% of the respondents reported that they had restless sleep often or all of the time. Consistent with previous research [13, 62, 63], we converted the measure into a dummy variable, assigning a score of 0 to those who none of the time or sometimes had restless sleep and a score of 1 to those who often or all of the time had restless sleep. This dichotomization approach can be further justified by an early study of Hankins [65], which found more reliable estimates and the least measurement error of this approach compared to other methods including the Likert method.

### Country-level predictors

A country’s level of gender equality is measured by the United Nation’s gender development index (GDI). The GDI measures gender gaps in life expectancy, education, and incomes by using the same component indicators as in the human development index (HDI). The GDI is the ratio of the HDIs calculated separately for women and men. It is a direct measure of gender gap showing the women HDI as a percentage of the men GDI. In line with previous research [13], we also applied per capita GDP to account for the relationship of economic development on sleep. To improve their interpretability, we multiplied the original GDI index by 100 and took the log transformation of per capita GDP.

### Individual-level predictors

#### Key individual predictor: Manager

The primary interest of this study was to explore whether those with greater workplace resources report more restful sleep. The ESS classified respondents’ occupations based on the International Standard Classification of Occupations 2008 (ISCO-08). We include a measure for whether the respondent is a **manager** (value = 1) or not (value = 0) as an occupational category to compare to those in other professional categories. In supplemental models, we also explored differences in sleep across a wider range of occupations (see S1 Table). Applying the ISCO-08, we classified occupations into 10 major groups: (1) managers; (2) professionals, technicians and associate professionals; (3) clerical support workers; (4) services and sales workers; (5) skilled agricultural; (6) forestry and fishery workers; (7) craft and related trade workers; (8) plant and machine operators and assemblers; (9) elementary occupations; and (10) armed forces occupations. We started by generating dummy variables for all occupational groups to explore the sleep effects of different occupations, but found limited evidence that sleep varied across these occupational groups. Thus, our modeling compares this group, identified as having the highest status, to all others.

In occupational literature, the definition of managers is broad including supervisors and team leaders. ISCO-08 highlights the distinction between managers and supervisors: while supervisors are responsible only for supervision of the activities of other workers, managers have overall responsibility for the operations of a business or an organizational unit. Further, managerial occupations are organized along functional rather than industrial lines. This means that managers with specialist functions are identifiable, irrespective of the industry in which they work [66].

#### Workplace controls

Respondents were asked to estimate the **total number of weekly hours** in their main job. Work time was top-coded and capped at 80 hours per week. Respondents were asked to what extent they were allowed to decide how daily work is organized (i.e., **daily work control**) and to what extent they were allowed to influence policy decisions about activities of their organization (i.e., **workplace policy control**). Responses are on a ten-point scale, with 0 having no influence and 10 having complete control.

#### Sociodemographic controls

We included a number of sociodemographic controls in our models shown to be associated with sleep [13]. We measured **age** using a series of dummy variables: 25 to 34, 35 to 44 (the reference group), 45 to 54, and 55 to 64. Respondents were asked to measure their **household’s total income** from all sources, which is standardized into deciles within countries to allow for cross-national comparability.

**Education** is measured on the EISCED a seven-point ordinal scale (1 = less than lower secondary, 7 = higher tertiary education, > = MA level) so higher values indicate higher educational attainment. The educational variable was regrouped into three dummies: college or above, sub-degree or upper secondary education (the reference group), and lower secondary education or below.

To control for family factors that could affect sleep, we included a binary measure of whether the respondent lives with a **husband/wife/partner**. We also include two dummies for the presence of children in the home: (1) **young child** (<6 years old) present (value = 1); and (2) **school-aged child** (6 to 17) present (value = 1), with the reference group consisting of childless respondents or those whose youngest child is an adult regardless of where s/he lived.

Since sleep is significantly influenced by health, we also included two key health-related items. **Poor self-reported general health** was assessed by responses on a five-point ordinal scale (1 = very good to 5 = very bad) to the question “How is your health in general?” To measure emotional well-being, ESS used six questions asking how often the respondents (1) felt sad, (2) felt depressed, (3) enjoyed life, (4) were happy, (5) felt anxious and (6) felt calm and peaceful in the past week [67]. Responses ranged from 1 = none of the time to 4 = all of the time. Items (3), (4), and (6) were reversed and the alpha coefficient of the six items was 0.79. The six measures were averaged to produce a single **poor emotional health index**, with higher scores indicating negative day-to-day feelings such as anxiety and depression, and lack of positive feelings such as happiness and enjoyment.

### Multilevel models

Due to the nested structure of the data, we analyzed restless sleep in multilevel regression models. Since we converted the dependent variable into a binary variable, we applied multilevel logit regression models. As a robustness test, we also estimated the models as ordinal logit regression and the results are substantively equivalent (results are presented in S2 Table). Both the GDI index and logged per capita GDP were centered on their respective grand means when entered into the country equations. Standard errors clustered at the country level. Each logit model was estimated separately by gender, and the significance of gender differences was tested in a pooled model.

## Results

### Descriptive statistics

Table 1 provides statistics of both the dependent variable and two key predictors at the country level. The second and third columns provide the proportions of women and men workers reporting restless sleep in 29 countries. In 27 out of 29 countries a larger proportion of women than men slept restlessly in the prior week (Estonia and Israel are the only two exceptions). The fourth column reports the (one-tailed) z-values of the difference between men’s and women’s reports of restless sleep; in 16 out of 29 countries the gender difference in restless sleep is statistically significant.

**Table 1:** Restless sleep by country and country-level predictors.

Country	Restless sleep, women	Restless sleep, men	z-values	GDI index	Per capita GDP, 2011 PPP
Albania	0.29	0.27	-0.36	0.972	10370
Belgium	0.19	0.15	-1.29*	0.970	41125
Bulgaria	0.12	0.08	-1.81**	0.991	15772
Switzerland	0.14	0.10	-1.60*	0.964	56150
Cyprus	0.20	0.10	-2.76***	0.974	31750
Czechia	0.17	0.14	-0.91	0.980	28527
Germany	0.21	0.15	-2.38***	0.963	42822
Denmark	0.14	0.10	-1.32*	0.981	44337
Estonia	0.12	0.13	0.73	1.023	25692
Spain	0.14	0.10	-1.74**	0.979	31109
Finland	0.09	0.07	-1.43*	1.005	39913
France	0.26	0.20	-2.16**	0.990	37377
United Kingdom	0.24	0.16	-2.68***	0.964	37094
Hungary	0.27	0.23	-1.07	0.990	22582
Ireland	0.10	0.09	-0.55	0.977	44829
Israel	0.11	0.12	0.47	0.971	30645
Iceland	0.15	0.10	-1.10	0.979	41077
Italy	0.14	0.12	-0.39	0.972	35228
Lithuania	0.08	0.05	-1.07	1.036	24049
Netherlands	0.14	0.11	-1.32*	0.967	45949
Norway	0.09	0.07	-1.35*	0.997	63003
Poland	0.18	0.11	-2.48***	1.009	23218
Portugal	0.09	0.06	-1.07	0.989	25806
Russian Federation	0.16	0.09	-2.60***	1.026	25156
Sweden	0.12	0.07	-2.65***	1.000	43356
Slovenia	0.13	0.10	-0.84	1.006	27977
Slovakia	0.09	0.09	0.20	0.987	26218
Ukraine	0.22	0.17	-1.33*	1.000	8322
Kosovo	0.06	0.06	-0.15	NA	NA
Correlation with GDI	-0.32*	-0.33*	--	--	--
Correlation with GDP	-0.31	-0.35*	--	-0.34*	--

Note:

*** p < .01,

** p < .05,

* p < .1. 1-tailed tests for gender differences in restless sleep.

The last two columns of Table 1 present the two country-level predictors included in our multilevel analyses. A quick reading of the figures reveals that Nordic countries generally rank high on gender development. Transition economies, especially those with a socialist legacy, also rank high on the gender development index. This likely reflects the legacy of socialism characterized by large investments in women’s employment and education [68].

The bottom two rows of Table 1 provide several correlation coefficients between the key variables. The correlation between restless sleep and GDI is -0.32 for women and -0.33 for men (both statistically significant at the 10% level), which provides some preliminary evidence that both women and men tend to sleep better in countries with higher GDI scores. The correlation between restless sleep and GDP is -0.31 for women and -0.35 for men. However, the correlation is only statistically significant for men but not for women, suggesting that men’s sleep is more responsive to economic growth, again lending some preliminary support for our hypothesized relationships (H2 and H3).

Table 2 summarizes the descriptive statistics of independent-level variables by gender. The right-most column shows the (one-tailed) z- or t-values for the difference between the men’s and women’s proportions/means on a given measure (two-sample tests of proportions for binary variables; ttests for continuous variables; and chi-square tests for categorical variables). The z scores/t values are negative when the proportion/mean for women exceeds that for men and are positive when the proportion for men exceeds that of women. Once again confirming our hypothesized relationship, men were less likely than women to report restless sleep. Men were more likely to work as a manager and their work time was significantly longer than that of women. Men also reported more control of their daily work and workplace policy.

**Table 2:** Descriptive data of individual-level variables.

	Min	Max	Proportion/mean for women	Proportion/mean for men	z-values	t-values	Chi-square
Having restless sleep	0	1	0.15	0.12	-7.38***	--	--
Key independent variables							
Being a manager	0	1	0.06	0.11	10.62***	--	--
Daily work control	0	10	6.69	6.96	--	--	55.75***
Workplace policy control	0	10	4.55	5.03	--	--	150.43***
Total hours worked in the past week	0	80	37.53	43.79	--	35.74***	--
Sociodemographic controls							
Age							
Between 25 and 34	0	1	0.22	0.23	2.78***	--	--
Between 35 and 44 (ref.)	0	1	0.29	0.29	-0.63	--	--
Between 45 and 54	0	1	0.30	0.28	-1.75**	--	--
Between 55 and 64	0	1	0.20	0.20	-0.20	--	--
Education							
College or above	0	1	0.36	0.29	-11.27***	--	--
Sub-degree or upper secondary (ref.)	0	1	0.52	0.57	7.40***	--	--
Lower secondary or below	0	1	0.12	0.14	4.74***	--	--
Household’s total net income (deciles)	1	10	6.11	6.42	--	--	92.69***
Living with partner	0	1	0.68	0.75	10.71***	--	--
Presence of child under six	0	1	0.14	0.17	5.81***	--	--
Presence of child between six and seventeen	0	1	0.35	0.31	-5.54***	--	--
Health and well-being							
Poor physical health	1	5	2.05	1.95	--	--	87.97***
Poor emotional health	1	4	1.85	1.74	--	--	261.50***

Note:

*** p < .01,

** p < .05,

* p < .1. 1-tailed tests.

### Multilevel models: Determinants of restless sleep

Table 3 shows the determinants of restless sleep by gender (pooled results are presented in S3 Table). Model A allows the intercepts to vary across countries, while Model B attempts to account for variation in the intercepts with GDI and logged per capita GDP as predictors. Model C explores cross-level interactions, and since the interpretation of the coefficients is slightly different compared to that for Models A and B, the models will be discussed separately.

**Table 3:** Multilevel logit models of restless sleep by gender.

	Model A		Model B		Model C		Significant gender difference
	Women	Men	Women	Men	Women	Men	
	N = 8,949	N = 9,167	N = 8,872	N = 9,002	N = 8,872	N = 9,002	
Key independent variables							
Being a manager	0.30***	-0.04	0.28**	-0.07	0.25**	-0.07	A, B
Total hours worked in the past week	-0.00	0.01***	0.00	0.01***	0.00	0.01***	**A, B**
Daily work control	0.02	0.02	0.02	0.02	0.02	0.02	
Workplace policy control	0.01	-0.02	0.007	-0.02	0.01	-0.02	
Sociodemographic controls							
Age: Between 25 and 34	0.19*	0.01	0.20*	-0.00	0.19*	-0.01	B
Age: Between 45 and 54	0.04	-0.03	0.05	-0.04	0.05	-0.04	
Age: Between 55 and 64	0.14	-0.09	0.16	-0.09	0.15	-0.09	
Education: College or above	-0.34***	-0.24**	-0.34***	-0.25***	-0.33***	-0.26***	
Education: Lower secondary or below	-0.06	0.18	-0.09	0.15	-0.09	0.15	
Household’s total net income	0.00	-0.01	0.004	-0.00	0.00	-0.00	
Living with partner	0.05	0.20**	0.05	0.20**	0.05	0.20**	
Presence of child under six	0.37***	0.31***	0.37***	0.33***	0.37***	0.33***	
Presence of child between six and seventeen	-0.18*	-0.06	-0.17*	-0.05	-0.17*	-0.05	
Health and well-being							
Poor physical health	0.40***	0.44***	0.41***	0.45***	0.41***	0.45***	
Poor emotional health	1.73***	1.72***	1.72***	1.72***	1.72***	1.72***	
Country equation, intercept							
General intercept	-6.32***	-6.81***	-6.31***	-6.82***	-6.30***	-6.81***	A, **B, C**
GDI	--	--	-0.14***	-0.16***	-0.13***	-0.16***	
Logged per capita GDP	--	--	0.007	-0.07	0.03	0.02	
Variance component intercept	0.02***	0.02***	0.01***	0.01***	0.01***	0.01***	
Cross-level interactions							
Managers * GDI	--	--	--	--	-0.09**	-0.01	
Managers * Logged per capita GDP	--	--	--	--	-0.35	-0.73***	

Note:

*** p < .01,

** p < .05,

* p < .1. Sample size for Models B and C differ from Model A because there’s no GDI and GDP data for Kosovo in the United Nation’s database. Country predictors are centered on their grand means. Standard errors clustered at the country level. In the last column, bolded letters represent statistical gender significance at the 5% level while normal letters represent statistical gender difference at the 10% level.

We found evidence that being a manager had a strong positive association with women’s restless sleep. In Models A and B, the coefficients on managers for women were 0.30 and 0.28 respectively and both coefficients were statistically significant. In contrast, the coefficients for men were negative, smaller in magnitude and not statistically significant. Our gender interactions models suggest these gender differences were statistically significant lending support to our gendered *stress of higher status* hypothesis (H1).

Model B tests our gender empowerment and economic development hypotheses (H2 and H3) for the entire sample. The results show that both men and women workers sleep better in countries with greater gender development, showing our gender empowerment hypothesis extends to men workers as well (H2). GDP, however, is not significant at the intercept, thus failing to support our economic development hypothesis (H3) for women and men workers.

Turning to other individual characteristics, we find men’s sleep seems to be more influenced by work hours than that of women. In both Models A and B, the coefficients on work time were statistically significant only for men and the gender difference was statistically significant. This can perhaps be attributed to the fact that men on average worked longer and thus experience a greater sleep disruption (6 hours/week longer than women, see Table 2).

We did not find evidence for stronger effects of the family-related variables on women’s sleep. In separate models, men reported more frequent restless sleep when living with a partner (b = 0.20, p < .05) while women tended to have better sleep with the presence of children aged 6 and 17 (b = -0.18, p < .1 in Model A and b = -0.17, p < .1 in Model B) compared to those without children. In addition, the sleep of both men and women was more restless with the presence of children aged under six. This finding differs from previous research [13] which found that only women’s sleep was more disturbed by the presence of young children, consistent with the cultural expectation that mothers are more accountable than fathers for the night-time care of young children. However, we note that our sample was restricted to working people only, thereby excluding women who quit the labor market to take care of their children. Thus, it is not surprising our results differ from this previous research, and they further suggest that women who maintain employment are equally disadvantaged in sleep to their men counterparts.

### Cross-level interactions

The results of Model B affirmed that women and men alike slept better when they lived in a more gender-equal society. We further investigated this relationship by hypothesizing that the country-level predictors may condition the effects of being a manager in predicting restless sleep (H2 and H3a; Model C). The results are also shown in Table 3.

Most results of Model C are similar to those of Models A and B. However, due to the inclusion of cross-level interactions, the interpretation of the coefficient on being a manager and country-level predictors changed. For women, the interaction between being a manager and GDI was negative and statistically significant, meaning that women managers reported more restful sleep in countries with higher GDI. For men, the interaction between being a manager and per capita GDP was negative and statistically significant, meaning that men managers were more likely to have better sleep in more economically developed countries.

**Fig 1:**
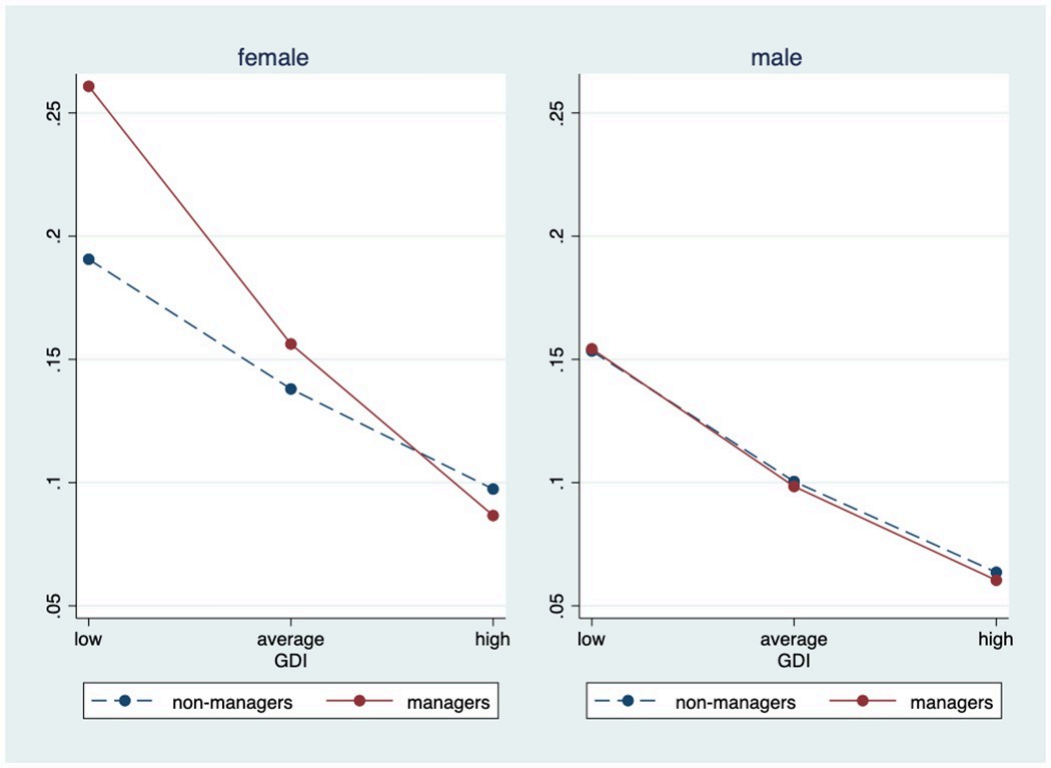
Predicted probability of restless sleep by gender development index score, (based on Model C).

**Fig 2:**
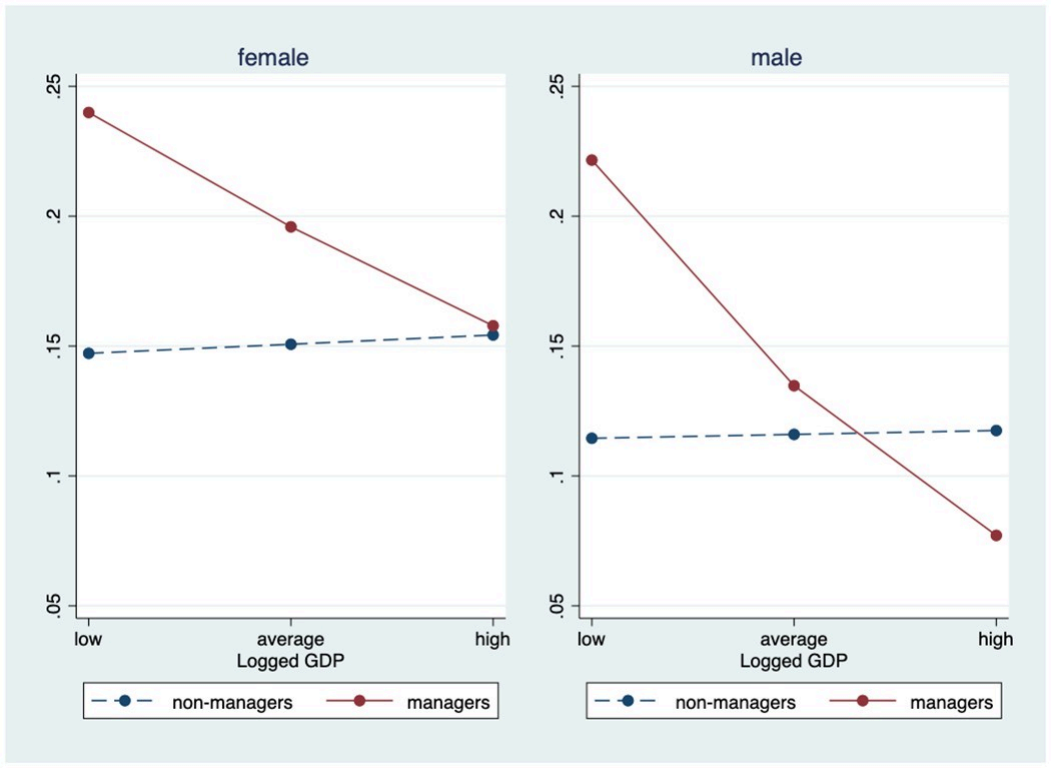
Predicted probability of restless sleep by logged per capita GDP (based on Model C).

To better interpret these results, the predicted probability of sleeping restlessly for managers and non-managers was estimated across the range of country-level predictors observed across Europe. To do this, we first calculated margins from predictions of Model C, at fixed centered values for minimum, average, and maximum values of GDI (minimum for Germany = -2.53 and maximum for Lithuania = 4.76). Then we estimated margins at fixed centered values for minimum, average, and maximum values of logged per capita GDP (minimum for Ukraine = -1.37 and maximum for Norway = 0.65). After calculating the predicted probabilities, we plotted the results in Figs 1 and 2.

Figs 1 and 2 show the results in a more intuitive way. As Fig 1 indicates, in countries of relatively low GDI, women managers were significantly more likely to report restless sleep, compared to employed women non-managers. However, in countries with high GDI, this difference was significantly narrowed and even reversed (support for H2). In other words, the effect of being a manager on women’s sleep is significantly conditioned by how well gender development is achieved in their country of residence. On the contrary, such a difference was not present for men. Although both men managers and employed men non-managers reported better sleep in more gender-equal societies, there was little difference between the two occupational groups.

The interaction between being a manager and per capita GDP is visualized in Fig 2. For both employed women and men non-managers, they reported slightly more frequent restless sleep in more economically developed countries. Managers had an opposite downward trend—their sleep improved with economic growth. This improvement was much more pronounced for men managers (support for H3a). The predicted probability for men managers in the country with the lowest level of per capita GDP (i.e., Ukraine) was 22%, a probability that was almost three times as large as men managers who lived in country at the highest level of per capita GDP (i.e., Norway). In highly economically developed countries, men managers had better sleep than men non-managers.

As a robustness check, we also tested whether the interaction of GDP and GDI structured sleep. The results were not significant, but we are wary of the impact of reduced statistical power through reduced degrees of freedom. Thus, we stress the importance of investigating these associations in future large surveys to provide clearer guidance on their interrelationship.

## Conclusion

The broad consensus in the health sociology literature is that higher social status is associated with better mental health outcomes through access to greater work resources [26]. Such a conventional view has been challenged by the stress of higher status theory, which highlights the increased level of stress exposure associated with higher-status positions. In particular, the literature documents that workers in such positions tend to experience higher levels of interpersonal conflict and work-to-home interference, which suppress the negative association between authority and poor health outcomes [23–25]. We extend this line of research to sleep, an important health outcome.

We investigated the relationship between sleep, managerial status and country-level economic and gender development. Our study found evidence corroborating the stress of higher status theory: compared to non-managers, managers were more likely to report restless sleep across Europe. However, we emphasize that this link between the managerial position and restless sleep is significantly structured by gender and economic development in ways that are distinctly gendered. Women managers report more restful sleep in countries with higher gender development. By contrast, men managers report more restful sleep in countries with stronger economies, or higher GDP. Collectively, our results indicate men managers experience a sleep premium from economic development and women managers from gender empowerment.

While we find good support for several of our hypothesis, there are a number of notable limitations to the study. Importantly, the cross-sectional design of ESS does not allow us to make causal claims. Although ESS conducts survey every two years, it selects new sample members each round. We see that economic development and gender empowerment play an essential role in shaping the sleep quality of working people but how this shift influences within-person change remains unclear. However, as no longitudinal panel on these topics exists for the population we studied, a cross-sectional investigation still makes a contribution by unveiling important patterns.

The use of a one item measure of sleep quality is also a limitation. Further, since the questionnaires were translated into different languages across countries, the wording of the item (sleep was restless) and interpretation may vary. However, these limitations are inevitable in large-scale cross-country surveys like the European Social Survey which employs advanced survey techniques to ensure validity across countries. Other measures of sleep may also be important, for example time spent in sleep, which is likely associated with time spent in work and managers often spend longer hours in work [2]. Applying time use data to investigate these relationships is a prime area for future research. As social scientists increasingly spotlight sleep as a form of inequality, a wider range of sleep measures should be included in large nationally representative surveys.

Our results present clear policy implications: economic growth alone is not enough—its sleep benefit is rather limited to men managers. By contrast, higher levels of gender development reduce the likelihood of having restless sleep, among men and women alike. In particular, a higher level of gender equality could improve women managers’ sleep, thereby reducing the stress of higher status experienced by women workers. In this way, our research adds to a previous study [13] by underscoring the potential benefits of policies aimed at reducing the human development gap between men and women.

## Supporting information

S1 Table Multilevel logit models of restless sleep by gender (including all occupational groups). (DOCX)

S2 Table Multilevel ordinal logit models of restless sleep by gender. (DOCX)

S3 Table Multilevel logit models of restless sleep. (DOCX)
