# Supplementary material for: Do managers sleep well? The role of gender, gender empowerment and economic development
Source: PLoS One. 2021 Mar 17;16(3):e0247515. doi: 10.1371/journal.pone.0247515 (PMC7968640; doi:10.1371/journal.pone.0247515)
Supplement: S1 Table — (DOCX) [file pone.0247515.s001.docx]

**S1 Table. Multilevel logit models of restless sleep by gender (including all occupational groups)**

|  | Model A’ | | Model B’ | | Model C’ | | Significant gender difference |
| --- | --- | --- | --- | --- | --- | --- | --- |
|  | Female | Male | Female | Male | Female | Male |  |
|  | N=8,949 | N=9,167 | N=8,872 | N=9,002 | N=8,872 | N=9,002 |  |
| Occupational groups |  |  |  |  |  |  |  |
| Managers | 0.35** | -0.11 | 0.32** | -0.13 | 0.29** | -0.12 | A, B |
| Professionals (ref.) |  |  |  |  |  |  |  |
| Technicians and associate professionals | 0.13 | -0.20* | 0.13 | -0.19 | 0.13 | -0.19 | A, B, C |
| Clerical support workers | 0.08 | 0.04 | 0.06 | 0.01 | 0.07 | 0.01 |  |
| Service and sales workers | 0.07 | -0.00 | 0.06 | -0.00 | 0.06 | 0.02 |  |
| Skilled agricultural workers | -0.38 | -0.36 | -0.38 | -0.34 | -0.38 | -0.32 |  |
| Craft and related trades workers | 0.05 | -0.07 | 0.01 | -0.05 | 0.01 | -0.04 |  |
| Plant and machine operators, and assemblers | 0.31 | 0.01 | 0.29 | 0.04 | 0.29* | 0.05 |  |
| Elementary occupations | 0.23 | -0.07 | 0.23 | -0.02 | 0.23** | -0.01 |  |
| Other key independent variables |  |  |  |  |  |  |  |
| Total hours worked in the past week | 0.00 | 0.01*** | 0.00 | 0.01*** | 0.00 | 0.01*** | **A, B** |
| Daily work control | 0.02 | 0.03 | 0.02 | 0.03 | 0.02 | 0.03 |  |
| Workplace policy control | 0.01 | -0.02 | 0.01 | -0.02 | 0.01 | -0.02 |  |
| Sociodemographic controls |  |  |  |  |  |  |  |
| Age: Between 25 and 34 | 0.20* | 0.02 | 0.21* | 0.00 | 0.20* | -0.00 | C |
| Age: Between 45 and 54 | 0.05 | -0.02 | 0.06 | -0.03 | 0.05 | -0.03 |  |
| Age: Between 55 and 64 | 0.15 | -0.08 | 0.17 | -0.09 | 0.16 | -0.09 |  |
| Education: College or above | -0.29*** | -0.26*** | -0.30*** | -0.27*** | -0.29*** | -0.28*** |  |
| Education: Lower secondary or below | -0.10 | 0.18 | -0.12 | 0.14 | -0.12 | 0.14 |  |
| Household's total net income | 0.01 | -0.01 | 0.01 | -0.00 | 0.01 | -0.00 |  |
| Living with partner | 0.05 | 0.20** | 0.04 | 0.20** | 0.05 | 0.20** |  |
| Presence of child under six | 0.37*** | 0.31*** | 0.37*** | 0.34*** | 0.37*** | 0.33*** |  |
| Presence of child between six and seventeen | -0.18* | -0.05 | -0.17* | -0.05 | -0.17* | -0.05 |  |
| Health and well-being |  |  |  |  |  |  |  |
| Poor physical health | 0.40*** | 0.44*** | 0.41*** | 0.45*** | 0.41*** | 0.45*** |  |
| Poor emotional health | 1.72*** | 1.71*** | 1.71*** | 1.71*** | 1.71*** | 1.72*** |  |
| Country equation, intercept |  |  |  |  |  |  |  |
| General intercept | -6.47*** | -6.78*** | -6.44*** | -6.81*** | -6.44*** | -6.82*** |  |
| GDI | -- | -- | -0.14*** | -0.16*** | -0.13*** | -0.16*** |  |
| Logged per capita GDP | -- | -- | 0.01 | -0.07 | 0.03 | 0.02 |  |
| Variance component intercept | 0.02*** | 0.03*** | 0.01*** | 0.01*** | 0.01*** | 0.01*** |  |
| Cross-level interactions |  |  |  |  |  |  |  |
| Managers * GDI | -- | -- | -- | -- | -0.09** | -0.01 |  |
| Managers * Logged per capita GDP | -- | -- | -- | -- | -0.36 | -0.74*** |  |

Note: *** p < .01, ** p < .05, * p < .1. Sample size for Models B’ and C’ differ from Model A’ because there's no GDI and GDP data for Kosovo in the United Nation’s database. Country predictors are centered on their grand means. Standard errors clustered at the country level. In the last column, bolded letters represent statistical gender significance at the 5% level while normal letters represent statistical gender difference at the 10% level.
