# Supplementary material for: Do managers sleep well? The role of gender, gender empowerment and economic development
Source: PLoS One. 2021 Mar 17;16(3):e0247515. doi: 10.1371/journal.pone.0247515 (PMC7968640; doi:10.1371/journal.pone.0247515)
Supplement: S3 Table — (DOCX) [file pone.0247515.s003.docx]

**S3 Table Multilevel logit models of restless sleep**

|  | Model A | Model B | Model C |
| --- | --- | --- | --- |
|  | N=18,116 | N=17,874 | N=17,874 |
| Key independent variables |  |  |  |
| Being a manager | 0.09 | 0.07 | 0.05 |
| Total hours worked in the past week | 0.00 | 0.00 | 0.00 |
| Daily work control | 0.02 | 0.02 | 0.02 |
| Workplace policy control | -0.01 | -0.01 | -0.01 |
| Sociodemographic controls |  |  |  |
| Age: Between 25 and 34 | 0.11 | 0.10 | 0.10 |
| Age: Between 45 and 54 | 0.02 | 0.03 | 0.03 |
| Age: Between 55 and 64 | 0.06 | 0.06 | 0.06 |
| Education: College or above | -0.27*** | -0.27*** | -0.27*** |
| Education: Lower secondary or below | 0.06 | 0.04 | 0.04 |
| Household's total net income | -0.00 | -0.00 | -0.00 |
| Living with partner | 0.10* | 0.10* | 0.10* |
| Presence of child under six | 0.35*** | 0.36*** | 0.36*** |
| Presence of child between six and seventeen | -0.11* | -0.10 | -0.10 |
| Health and well-being |  |  |  |
| Poor physical health | 0.42*** | 0.42*** | 0.42*** |
| Poor emotional health | 1.74*** | 1.73*** | 1.72*** |
| Country equation, intercept |  |  |  |
| General intercept | -6.49*** | -6.47*** | -6.46*** |
| GDI | -- | -0.14*** | -0.14*** |
| Logged per capita GDP | -- | -0.04 | 0.02 |
| Variance component intercept | 0.03*** | 0.01*** | 0.01*** |
| Cross-level interactions |  |  |  |
| Managers * GDI | -- | -- | -0.05** |
| Managers * Logged per capita GDP | -- | -- | -0.59*** |

Note: *** p < .01, ** p < .05, * p < .1. Sample size for Models B and C differ from Model A because there's no GDI and GDP data for Kosovo in the United Nation’s database. Country predictors are centered on their grand means. Standard errors clustered at the country level.
